# Systematic Review of Interventions to Improve the Provision of Information for Adults with Primary Brain Tumors and Their Caregivers

**DOI:** 10.3389/fonc.2015.00001

**Published:** 2015-01-23

**Authors:** Danette Langbecker, Monika Janda

**Affiliations:** ^1^School of Nursing, Institute of Health and Biomedical Innovation, Queensland University of Technology, Brisbane, QLD, Australia; ^2^School of Public Health and Social Work, Institute of Health and Biomedical Innovation, Queensland University of Technology, Brisbane, QLD, Australia

**Keywords:** neuro-oncology, brain tumor, information, doctor–patient communication, caregivers

## Abstract

**Background:** Adults with primary brain tumors and their caregivers have significant information needs. This review assessed the effect of interventions to improve information provision for adult primary brain tumor patients and/or their caregivers.

**Methods:** We included randomized or non-randomized trials testing educational interventions that had outcomes of information provision, knowledge, understanding, recall, or satisfaction with the intervention, for adults diagnosed with primary brain tumors and/or their family or caregivers. PubMed, MEDLINE, EMBASE, and Cochrane Reviews databases were searched for studies published between 1980 and June 2014.

**Results:** Two randomized controlled, 1 non-randomized controlled, and 10 single group pre–post trials enrolled more than 411 participants. Five group, four practice/process change, and four individual interventions assessed satisfaction (12 studies), knowledge (4 studies), and information provision (2 studies). Nine studies reported high rates of satisfaction. Three studies showed statistically significant improvements over time in knowledge and two showed greater information was provided to intervention than control group participants, although statistical testing was not performed.

**Discussion:** The trials assessed intermediate outcomes such as satisfaction, and only 4/13 reported on knowledge improvements. Few trials had a randomized controlled design and risk of bias was either evident or could not be assessed in most domains.

## Introduction

The provision of appropriate and timely information, tailored to the medical condition, needs, and preferences, is essential to allow patients and their families to cope with the diagnosis, access supportive resources, and reduce uncertainty and distress (1, 2). Information provision is essential for participation in decision-making and to enable patients to give informed consent for treatment (3–5). It also may improve compliance with treatment (6). Both during and after active treatment, information can aid patients and their families to monitor symptoms and undertake self-care. Information can also assist family members to develop skills to undertake caring tasks (7–9).

A range of strategies have been developed to facilitate information provision in the cancer setting more widely. Traditional approaches include written information, videos, CD or, more recently, websites and apps (10, 11). Strategies may also aim to improve communication between patients and healthcare professionals by means of treatment summary letters, provision of audio-tapes of consultations to patients, and communication skills training for doctors. Overall, these have been developed and evaluated widely for patients with cancer. Promising findings have specifically been shown with the use of such strategies for those with high needs, including patients requiring treatment for lung cancer or palliative care, with promising findings (12, 13).

Specifically for patients with brain tumors, studies suggest that they are not satisfied with the information that they have been provided. Patients want to receive more information, and wish the information to be more detailed (14–22). Further information is particularly required in two areas: (1) fatigue, insomnia, and psychological disturbance (17, 23); and (2) changes in physical function and body image (24). Caregivers require information on how to provide care (25), and how to manage physical, cognitive, and personality changes in the patient and cope with changes in family roles (26). The reasons why these needs are not well met are not clear; however, certain factors are apparent. In terms of patient characteristics, distress resulting from the diagnosis may impair some patients’ abilities to process information, particularly as the brain is commonly understood to define the “self” (27). Cognitive and physical changes resulting from a brain tumor or its treatment may also impair information seeking or comprehension for some patients (28). Cognitive impairment is the most common deficiency in primary brain tumor patients, particularly affecting executive function, visuoconstructive abilities, attention, and verbal memory (29). Memory loss, information processing, and attention are commonly affected by radiotherapy and chemotherapy (30). Deficits may also arise due to the tumor itself, raised intracranial pressure, or as the result of surgery (31). Cognitive impairment has been shown to affect patients’ awareness of their prognosis and ability to process information (32). Considering factors relating to healthcare professionals, the information provided may be insufficient due to clinicians’ views of what patients need. For example, some healthcare professionals may hold back “unnecessary” information in an attempt to “protect” patients from distress, particularly with regard to issues such as preparing wills, advanced health directives, or the immediacy of palliative care required (33). Materials used to convey information also have limitations, as they often require higher levels of literacy than is common in the population (34).

Patient, healthcare professional, and interactive issues are also likely to impact how well interventions aiming to improve information provision will reach patients with brain tumors and improve their satisfaction with care. Although some (but by no means all) informational interventions have been well studied in general cancer populations, the cognitive impairments experienced by brain tumor patients and the resulting concerns of this group are unique, and it cannot be assumed that interventions will be equally effective when applied to these patients and their caregivers. This review thus aimed to examine whether patient-, caregiver-, or healthcare professional-directed interventions improve information provision, satisfaction with the intervention, or other commonly assessed outcomes (35) such as knowledge, understanding, or recall for adults diagnosed with primary brain tumors and/or their family or caregivers.

## Materials and Methods

### Criteria for considering studies for this review

Randomized and non-randomized trials including single arm studies were eligible for inclusion. To be included, studies needed to test one or more interventions, which tested an educational component (i.e., involving knowledge transfer, using any format or materials) and which reported one or more of the outcomes: information provision, knowledge, understanding, recall, or satisfaction with the intervention. There were no language restrictions. Case reports, personal narratives, editorials, commentaries, and reviews were excluded.

As this review was concerned with outcomes for adults diagnosed with primary brain tumors and/or their informal caregivers, studies with both adults and children need to report outcomes for adults (18+ years) and children (<18 years) separately, or at least 75% of the sample needed to be aged 18+ years. Similarly, at least 75% of patients needed to be diagnosed with primary (malignant or benign) brain tumors, or outcomes needed to be reported for primary brain tumor patients separately. Studies involving caregivers were eligible either in conjunction with or separately to studies involving patients. Caregiver studies were eligible only for informal or family caregivers (i.e., not paid caregivers or healthcare professionals), although studies involving interventions targeting healthcare professionals were eligible where the aim of the intervention was to ultimately improve information provision to primary brain tumor patients or caregivers.

### Search methods for identification of studies

Searches of PubMed, MEDLINE, EMBASE, CINAHL (via EBSCOhost), and PsychINFO (via EBSCOhost) were conducted for the years 1980–2014, to identify reports of relevant studies. Search terms used medical subject headings (MeSH) and keywords relating to brain tumors, patient education, doctor–patient communication, and information provision (see Box 1 for an illustration). We also reviewed the reference lists of included studies and relevant reviews for further references to relevant trials.

Box 1 **Search terms used for MEDLINE.**

**Table:** 

Search	Query content
S1	Brain neoplasms (MeSH)
S2	Neuro-oncology OR neuro-oncology (title/abstract)
S3	Glioma OR glioblastoma OR astrocytoma OR meningioma OR schwannoma OR oligodendroglioma OR medulloblastoma OR ependymoma (title/abstract)
S4	Brain tumor OR brain tumor OR brain cancer OR brain neoplasm (title/abstract)
S5	1 OR 2 OR 3 OR 4
S6	Patient Education as Topic (MeSH)
S7	Professional Patient Relations (MeSH)
S8	Information Dissemination (MeSH)
S9	Consumer Health Information (MeSH)
S10	Pamphlets (MeSH)
S11	Audiovisual aids (MeSH)
S12	Information provision (title/abstract)
S13	6 OR 7 OR 8 OR 9 OR 10 OR 11 OR 12
S14	5 AND 13
S15	Animals NOT humans (MeSH)
S16	14 NOT 15
S17	Limit date 1980–June 30 2014

### Data collection and analysis

Articles identified from all sources were downloaded into a reference management software package and duplicates were removed. One author pre-screened all results (titles and abstracts) for possible inclusion based on the inclusion criteria. The full text of selected articles was then obtained and assessed against the inclusion criteria. Data were extracted by one author using a template, collecting study design, population, intervention characteristics, and outcomes. Where data were missing or unclear, or to obtain additional data, we attempted to contact lead study authors, to obtain the data needed for analyses. Where necessary, titles, abstracts, and full text were translated into English to allow assessment and data collection.

Both authors independently assessed risk of bias in individual studies in seven domains (random allocation sequence generation, allocation concealment, blinding of participants and personnel, blinding of outcome assessment, incomplete outcome data, selective outcome reporting, and other sources of bias), taken from the Cochrane Handbook of Systematic Reviews (36). Non-randomized and single arm studies were assessed and reported as being at a high risk of bias on the random allocation sequence generation and allocation concealment items of the “Risk of bias” tool. Risk of bias ratings was compared and consensus reached.

We had planned to pool the data across studies statistically using meta-analysis but the heterogeneity in intervention types, outcomes, and study designs meant that the data were unsuitable for this. We have thus conducted a narrative synthesis of results, grouping the data based on the category that best explores the heterogeneity of studies, in this case nature of the intervention (group level, practice or process change, or individual level). Within each category, we narratively summarized the results.

## Results

### Search results

**Figure 1:**
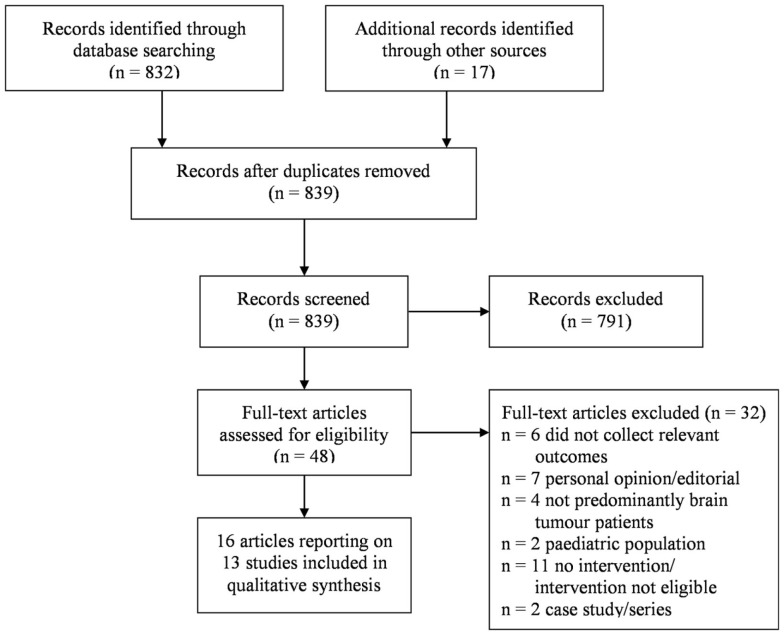
**Inclusion/exclusion process following article search**.

Eight hundred and thirty-nine original articles were identified, 48 of which were assessed at the full text level for eligibility. The screening and selection process is outlined in a PRISMA flow chart, see Figure 1.

### Included studies

A total of 16 articles reporting on 13 studies involving more than 210 patients, 87 caregivers and 104 healthcare professionals were selected for inclusion (Tables 1 and 2). Studies for which quantitative data were available are described in Table 1; Table 2 reports on the studies for which no quantitative results were reported. Two studies were randomized controlled trials (37, 38), one was a non-randomized trial with control group (39), and the remainder was single arm trials (40–48). Studies were most commonly conducted in the US [6 studies (38, 41–44, 46)] and Australia [3 studies (39, 47)], with single studies conducted in Canada (40), Austria (45), the Netherlands (37), and the UK (48). Six studies involved patients only (37–39, 41, 42, 48), two targeted caregivers only (43, 47), two healthcare professionals only (44, 47), and two both patients and caregivers (45, 46). A single study reported patient and healthcare professional participants (40), although only the patient participants were eligible for and included in this review. Four studies did not specify the sample size (43, 48), or data collection was in progress at the time of reporting (37, 42). Median participant sample sizes were 32 (range 13–50) for patients, 39 (range 7–41) for caregivers, and 52 (range 43–61) for healthcare professionals. One study was published only as a protocol (37), and four studies only in conference abstracts (41–43, 46). An attempt was made to contact corresponding authors of all included studies in order to verify methods and to obtain missing data, and six authors responded to requests for additional information.

**Table 1:** **Characteristics of included studies reporting quantitative results**.

Study (country)	*N*	Setting and participants	Intervention characteristics and comparison	Outcomes of interest and measures	Reported findings according to authors
**RANDOMIZED CONTROLLED TRIALS**					
El-Jawahri et al. (38, 51) (US)	50	Consecutive patients with malignant glioma, recruited via hospital oncology outpatient clinics	Video after verbal narrative, describing three levels of medical care in advanced cancer (life-prolonging care, basic medical care, comfort care). Six minute video shown on portable computer included visual images of the goals of care described verbally. Comparison: verbal narrative only	Knowledge (goals of care options assessed via questionnaire, yielding score 0–6). Patient satisfaction (perceived value of video, three items on 4-point Likert scale) assessed for intervention group only, immediately after intervention	Significantly higher mean increase in knowledge score for intervention (mean 1.9, 95% CI, 1.3–2.4) than control group (mean 0.9, 95% CI, 0.4–1.3), *p* = 0.004. Most intervention participants were “very comfortable” watching the video (82.6%), found it “very helpful” (78.3%), and would “definitely recommend it” (82.6%)
**NON-RANDOMIZED TRIALS WITH CONTROL GROUP**					
Langbecker et al. (39, 49, 50) (Australia)	20	Primary brain tumor patients diagnosed in previous 6 months and/or undergoing treatment, recruited via four hospitals	Brain tumor-specific question prompt list (booklet with list of questions patients may wish to ask) designed to facilitate patient-HCP communication with questions about: diagnosis; prognosis; symptoms and changes; treatment; support; after treatment finishes; the healthcare professional team. Control participants given standard brochure only	Quantity and quality of information received (assessed using EORTC QLQ-INFO25 questionnaire); satisfaction (acceptability of the intervention or standard brochure assessed using 17 questions, combined into summative index), collected 4–6 weeks after intervention	Higher median change in information received for intervention (2.7, range −24.0 to 18.6, *n* = 9) than control group (−2.0, range −36.0 to 9.3, *n* = 8), indicating greater information received. Median acceptability score higher for intervention (31, range 27–34) than control group (28, range 15–31), indicating greater acceptability
**SINGLE ARM STUDIES**					
Grimes (48)(UK)	NS	Patients with brain tumors using a neurosciences service at a hospital	(1) New package of patient admission process documentation covering issues to discuss with/communicate to patients at appropriate points during their stay; (2) procedures to reduce time waiting for biopsy result and for nurse to coordinate meeting to delivery results to patient; (3) communication training programs for staff; (4) information to familiarize patients with the hospital and covering types of diseases, treatments, and support services	Patients’ views on clarity of explanation, collected via survey using visual analog scales following patients’ receipt of their biopsy results. Collected prior to and 6 months after implementation of intervention	At baseline, 48% rated clarity of explanation; this was 73% after intervention (no data supplied to interpret result)
Delaney et al. (40) (Canada)	13	Consecutive newly diagnosed HGG patients undertaking chemoradiotherapy at a neuro-oncology outpatient clinic	Pharmacist integration into multi-disciplinary team. Initially took medication history and provided counseling re: chemotherapy administration; side effect management; dosing of supportive medications; drug interactions; communication with pharmacists; other medication-related questions. Called patient the next day and 5 days after treatment initiation to address medication-related questions and review treatment protocols; patient could also initiate contact	Patient satisfaction (perceptions of the pharmacist and benefit of their involvement in their healthcare team), collected at the end of the 3-month study	11/11 participants reported receiving useful information from pharmacist; 8/10 felt pharmacist’s presence was helpful in their initial consultation; 7/10 said pharmacist’s call on day 5 of treatment was useful; 8/10 said pharmacist answered additional drug-related questions to their satisfaction; 9/10 recommend pharmacist remains part of team
Green et al. (41) (US)	38	Patients with primary brain tumors living regionally from a Neuro-oncology Center	Use of a videoconferencing system for neuro-oncology follow-up visits, involving history-taking, physical examination, desktop sharing of clinical and laboratory data using an electronic medical record, sharing of neuro-images	Patient satisfaction (16 question online survey), timing unclear	Average level of satisfaction reported by patients was 9.8 (1–10 scale, SD not reported)
Rabow et al. (44) (US)	61	Neurosurgeons, neuro-oncologists, and other clinicians from a neurological surgery or integrated medicine department or attending national conferences	48 min documentary film entitled “The Caregivers” depicting stories of four family caregivers of adults with brain tumors and designed to improve neurosurgery training around supporting family caregivers. Screenings held for staff and at conferences	Satisfaction (perceived quality of the film, perceived importance; belief they learned something from the film, believe that the film was an effective way to teach about family caregivers, belief that the film should be seen by all clinicians caring for patients with brain tumors, collected on 10-point Likert scale) immediately after screening	Mean scores: 9.27 for quality; 9.03 for importance; 9.67 for learning something new; 8.98 for the film being an effective way to teach; 9.23 for the film should be seen by all clinicians (SDs not reported)
Schratter-Sehn et al. (45) (Austria)	104	Patients with high-grade glioma (glioblastoma, mixed glioma and astrocytoma) and their relatives recruited through neuro-oncology ward at hospital	Interdisciplinary group intervention led by a psychologist and physician, offered monthly, for participants to receive or exchange information. Flexible group therapy with 6–10 participants covering up to 2 therapy units (1.5 h). Aims: to be responsive to each participant’s needs and develop coping strategies, based on principle of “care, encourage, inform, and guide”	Satisfaction (how much participants liked the intervention) assessed via questionnaire, timing unclear	92% of participants said the intervention provided a context in which they could openly talk about their anxieties, concerns and needs. 93% indicated their questions were answered through the intervention. Requirements and expectations were met for 82% of patients and 78% of relatives
Whiting et al. study 1 (47, 52) (Australia)	7	Family caregivers of adult primary brain tumor patients who had participated in previous descriptive study	Half-day didactic workshop delivered by multi-disciplinary team to train family members in compensatory strategy use to manage challenging behaviors (reasons for, types of and strategies for managing behavioral and cognitive changes). Caregivers and patients attended sessions together with clinician-facilitated discussion	Knowledge and use of compensatory strategies, measured via Strategy Use Measure (SUM-Family), a 9-item Likert-type scale; satisfaction (usefulness of each workshop section) assessed via questionnaire immediately after workshop	Median SUM-Family global scores significantly increased from before (3.29, IQR = 0.80) to after (3.86, IQR = 0.81) the intervention, *p* < 0.05. Average rating of the workshop was 4.73 (4 = good; 5 = very good)
Whiting et al. study 2 (47, 52) (Australia)	43	HCPs recruited via professional networks	6 h workshop delivered by multi-disciplinary team including didactic presentations and small-group exercises covering the journey of a brain tumor patient, description of challenging behaviors and prevalence following brain tumor; principles of behavior management; case study and group activity	Knowledge of compensatory strategies measured via Strategy Use Measure (SUM), a 16-item Likert-type scale developed for study); satisfaction (evaluation of all sections of workshop) assessed immediately after workshop	Average SUM rating scores significantly increased from before (3.17) to after (4.1) the intervention (SDs not provided, paired *t*-test *p* < 0.001). Satisfaction mean scores were ≥ 4 (4 = good; 5 = very good)

*HCP, healthcare professional; CNS, central nervous system; NS, not specified; EORTC QLQ-INFO25, European Organisation for Research and Treatment of Cancer Information module; SD, standard deviation; IQR, interquartile range*.

**Table 2:** **Characteristics of included studies not reporting quantitative results**.

Study (country)	*N*	Setting and participants	Intervention characteristics and comparison	Outcomes of interest and measures	Reported findings according to authors
**RANDOMIZED CONTROLLED TRIALS**					
Boele et al. (37) (The Netherlands)	NS	Adult grade II, III or IV glioma patients with mild-moderate depressive symptoms and their informal caregivers, recruited through advertising and treating HCPs	Internet-based self-help course based on principles of problem solving, with information about specific diseases and treatment, and psychological impact on everyday life. Five modules (text and exercises), 2 h/week over 5 weeks. Feedback from personal coach. Wait list control and non-CNS malignancy control group	Satisfaction (usability, readability, usefulness of the course and coach’s feedback assessed by questionnaire) immediately and 6 months after intervention	Data collection in progress
**SINGLE ARM STUDIES**					
Lima et al. (42) (US)	NS	Newly diagnosed primary brain tumor patients at a Comprehensive Cancer Center	Survivorship care delivery model involving nurse practitioner survivorship visits in coordination with primary neuro-oncologist. Aims: to identify and manage symptoms and distress; patient education; facilitation of communication among care providers; navigation of resources. Visits scheduled within 3 weeks of diagnosis and at specific points in the disease trajectory. Included personalized education notebook, calendar, pedometer, and “walking challenge,” after visit summary and written summaries sent to all treatment team members	Satisfaction regarding initial survivorship visit and patient education notebook (collected by survey), timing unclear	Data collection in progress
Patterson and Lovely (43) (US)	NS	Family caregivers of brain tumor patients, implemented at medical centers	8-h workshop curriculum providing information on topics such as medical overview of brain tumors, symptom management at home, understanding cognitive changes, how to safely move a patient. Offered by oncology nurses and aims to develop practical care skills	Caregiver knowledge (measured by questionnaire), satisfaction (overall benefit of the workshop as perceived by participants), timing unclear	No results reported
Spezeski et al. (46) (US)	75	Callers to a neuro-oncology telephone service (35% patients, 52% family/friend of patient)	Neuro-oncology information telephone line providing information on topics such as brain tumor types and treatments, caregiving issues, symptom management, and referrals to support-related resources	Satisfaction (measurement tool unclear)	“Callers expressed satisfaction with their experience and found the information to be quite helpful” (p. 549). “Virtually all callers said they would recommend the hotline to others needing information about brain tumors” (p. 549)

*HCP, healthcare professional; NS, not specified*.

Five interventions were delivered at the group level, four intervened to facilitate practice or process changes, and four were individual level interventions. At the group level, two workshops provided training in using compensatory strategies to manage challenging behaviors; one half-day duration workshop delivered by a multi-disciplinary group covering didactic sessions and clinician-facilitated discussions was for family members (47) and the other 6-h workshop, also led by a multi-disciplinary team and involving didactic presentations and small-group exercises, was for healthcare professionals (47). A further workshop of 8 h duration provided training for family members to develop practical care skills and provide information about brain tumors (43). Schratter-Sehn (45) and colleagues described a mixed patient/family member information and support group, which took place monthly with flexible attendance. Rabow and colleagues (44) developed and screened a 48 min documentary film for neurosurgeons, neuro-oncologists, and other clinicians to teach them about family caregiving for patients with brain tumors.

At the practice or process level, Lima and colleagues (42) described an evaluation currently in progress of a new survivorship care model involving nurse practitioner survivorship visits in coordination with neuro-oncologists. This intervention includes scheduled survivorship visits, a personalized education notebook, calendar, pedometer, and “walking challenge,” electronic medical record-created “After Visit Summary” and written summaries sent to all treatment team members. Delaney and colleagues (40) described the integration of a pharmacist into the neuro-oncology team, with the pharmacist meeting with or telephoning patients three times during their course of chemotherapy, and returning patient-initiated calls during this time. Pharmacists provided standardized counseling regarding chemotherapy administration, managing side effects, dosing of supportive medications and drug interactions, and communication with pharmacists, and answered other medication-related questions. Green and colleagues described the use of a videoconferencing system to allow brain tumor patients to undergo follow-up neuro-oncology visits at a medical center closer to home, rather than having to attend a tertiary hospital further away. Following the taking of history and physical examination, clinical and laboratory data and neuro-images were shared by desktop by a neuro-oncologist located at the tertiary center. Finally within this category, Grimes and colleagues described the evaluation of changes to a number of processes within a hospital neurosciences service. Changes included documentation for staff relating to the patient admission process; training programs for staff relating to the communication of “bad news” to patients; documentary information for patients and families covering types of disease, treatment, and support services; new systems for the management of scans and biopsy results; and a half hour preparation session for patients held at the beginning of each neuro-oncology clinic, during which patients were allocated to a single clinician based on their needs (rather than seeing each clinician as done previously).

At the individual level, a wide variety of interventions were evaluated. El-Jawahri and colleagues (38) tested a 6-min video designed to facilitate end-of-life discussions in a randomized controlled trial. The video depicted life-prolonging care [for example, including cardiopulmonary resuscitation (CPR), intubation and mechanical ventilation], basic care (including hospitalization, intravenous fluids, and antibiotics but excluding CPR, etc.) and comfort care (usually including medications to improve symptoms but not hospitalization). Boele and colleagues (37) described a self-administered internet-based intervention based on problem-solving therapy for glioma patients with mild to moderate depressive symptoms. The intervention consisted of five modules with text and exercises, with feedback provided by a personal coach. Spezeski and colleagues (46) described the evaluation of a neuro-oncology information hotline, which patients and caregivers could call as desired and which covered topics ranging from brain tumor types and treatments, caregiving issues, symptom management, and referrals to support-related resources. Langbecker and colleagues (39) tested a brain tumor-specific question prompt list, which is a structured list of questions for patients to ask of healthcare professionals if they wish and which may foster the provision of tailored, personally relevant information.

The most commonly reported outcome was satisfaction with the intervention, assessed in some form (e.g., found the intervention helpful or acceptable) by 12 of the 13 studies (37–47, 49–52). Four studies assessed knowledge by questionnaire (38, 43, 47) in terms of knowledge of different levels of medical care in the advanced stage of cancer (38), knowledge and use of compensatory strategies to manage behavioral and cognitive changes (47), or caregiver knowledge not further defined (43). Two studies assessed information provision (39, 48). No studies assessed recall or understanding, and only two studies assessed more distal outcomes such as quality of life (37, 39). Outcomes were most commonly assessed immediately after the intervention (37, 38, 44, 47), although the timing of assessment was not clear in six studies (41–43, 45, 46, 48).

### Risk of bias in included studies

Both randomized controlled trials (37, 38) were rated as low risk for random sequence generation and allocation concealment (Table 3). As all other studies were non-randomized or single arm studies, risk was rated as high for these biases. Blinding of outcome assessment was rated only for the three studies with control groups, and was rated as high for all three (37, 38, 49) as well as all single arm studies due to the nature of the interventions. Five studies (38–40, 44, 47) were rated as low risk with regard to incomplete outcome data, with all other studies rated as unclear risk due to absence of a published protocol. Only two studies (39, 44) were rated as low risk for selective reporting, with corresponding authors confirming that all outcomes were reported.

**Table 3:** **Risk of bias for included studies**.

Study	Random sequence generation	Allocation concealment	Blinding of outcome assessment	Incomplete outcome data	Selective reporting
Boele et al. (37)	Low	Low	High	Unclear^a^	Unclear^a^
El-Jawahri et al. (38, 51)	Low	Low	High	Low	Unclear
Langbecker et al. (39, 49, 50)	High	High	High	Low	Low
Grimes (48)	High	High	High	Unclear	Unclear
Delaney et al. (40)	High	High	High	Low	Unclear
Green et al. (41)	High	High	High	Unclear	Unclear
Lima et al. (42)	High	High	High	Unclear^a^	Unclear^a^
Patterson and Lovely (43)	High	High	High	Unclear	Unclear
Rabow et al. (44)	High	High	High	Low	Low
Schratter-Sehn et al. (45)	High	High	High	Unclear	Unclear
Spezeski et al. (46)	High	High	High	Unclear	Unclear
Whiting et al. study 1 (47, 52)	High	High	High	Unclear	Unclear
Whiting et al. study 2 (47, 52)	High	High	High	Low	Unclear

*^a^Unclear risk as data collection in progress*.

### Effects of interventions

The effects of interventions are reported only for the studies described in Table 1, for which quantitative results are available. Where appropriate, we have highlighted where studies assessed outcomes but did not report the results of these outcomes.

#### Outcome: information provision

One non-randomized study and one single arm study assessed information provision (39, 48). Grimes (48) compared the views of inpatients on the clarity of information provided to them before and after intervention implementation. Patient-reported clarity of explanation increased from 48 to 73% after the intervention (no information was provided to explain how to interpret these percentages). Langbecker and colleagues (39) assessed the quality and quantity of information received by participants using the European Organisation for Research and Treatment of Cancer (EORTC) information module (QLQ-INFO25). In a non-randomized trial, the median change in information received between baseline and follow-up was higher for intervention group participants (a brain tumor-specific question prompt list) compared to brochure only controls. However, statistical testing of the significance of these group differences was not reported, and the sample size was small, with follow-up data collected for 17 of 20 participants only due to attrition. Overall, both studies showed that greater information was provided to participants who received the interventions compared to those who did not, although the high risk of bias for both studies for randomization, allocation concealment, and blinding of outcome assessment limits the confidence that can be had in these findings.

#### Outcome: knowledge

One randomized controlled trial (38) tested a video and three single arm pre-/post-test studies (43, 47) tested the effect of workshop-delivered interventions on participants’ knowledge; however, no results were reported for one study, which evaluated the effect of a workshop for family members (43). Among the three studies for which results were available, the randomized controlled trial showed a statistically significantly greater mean increase in patients’ knowledge of the different levels of medical care in the advanced stages of cancer for patients who received the video intervention compared to those who received the control condition (38). Compared to pre-workshop levels, Whiting and colleagues’ (47) interventions led to statistically significantly increases in participant knowledge (for family members and for healthcare professionals) following the workshops.

Although these results are promising, study-specific instruments were used to assess knowledge for all three of these studies (38, 43, 47). Whiting et al. (47) reported that the instrument (the Strategy Use Measure) used to assess knowledge for healthcare professionals (and a modified version of this was also used to assess knowledge for family members) showed strong internal consistency and did not demonstrate ceiling or floor effects (47). While this psychometric information demonstrates reliability, the validity of the instrument and its sensitivity to change is unclear.

The contextual significance of these results is also unclear. Statistical significance may be shown with a large enough sample even if the clinical or contextual significance of the findings is unremarkable. However, the sample sizes of three studies were small, including 50 patients (38), 7 family members (47), and 43 healthcare professionals (47). The presence of statistically significant results with such small samples provide support for the significance of the results, but further research to validate the instruments and establish the significance of different levels of change is needed.

Risk of bias was not significantly different across the three studies for which data were available, so sub-analysis of the impact of risk of bias was not possible.

#### Outcome: satisfaction

Twelve studies (37–47) considered satisfaction with the intervention as an outcome, and nine studies reported (38–41, 44–47) data relating to this outcome, primarily described as the intervention’s acceptability, perceived usefulness, value, or quality. Among the nine studies for which results were available, only one study reported comparative data for intervention and control groups; Langbecker et al. (39) reported that a greater proportion of participants who received a question prompt list compared to those who received a control brochure highly agreed that the brochure was helpful, assisted them to ask questions, and other satisfaction items. All other studies reported satisfaction in intervention group participants only. They found high rates of satisfaction, evidenced by mean satisfaction scores of at least 8 out of 10 (or equivalent), or at least 80% of participants selecting the highest rating on a Likert-type scale. This was true regardless of the nature of the intervention, whether it was delivered in a group or individual setting, or constituted a practice or process change, and regardless of the risk of bias of the studies involved.

## Discussion

Our findings suggest that if an intervention is provided to patients with brain tumors, their caregivers, or the healthcare professionals who treat these patients, satisfaction ratings improve. These findings are based largely on non-randomized pre–post single arm intervention studies, mostly with relatively small sample sizes. Although similar to reports from previous reviews in the wider cancer population, the analyses focusing on those affected by brain tumors reported here provide additional insight. First, the review provides evidence for the feasibility of conducting studies with this patient and caregiver population. This is important as some may doubt that the highly distressed and often cognitively impaired population may be willing to be included in such investigations. Based on this review’s findings, those who agree to participate can be reassured that they will benefit at least subjectively. The reviewed studies also provide suggestions for optimizing data collection in the brain tumor patient population to reduce study burden, such as collecting data immediately after the intervention (38) or collecting data by interview rather than self-administered forms (39). Both of these strategies are recommended for palliative care research and may have value in this population (53).

However, the review also highlights a lack of stringent outcome measurements, which can be compared across studies or can be objectivized. This could include standardized tests of knowledge or improvements in treatment compliance, which often are target aims, but were seldomly formally assessed. Notable exceptions are the study by El-Jawahri et al. (38), who used a standardized knowledge score as outcome measure, and Langbecker et al. (39), who used an EORTC module to assess improvements in information. The most appropriate outcomes to measure in future studies must also be considered. Satisfaction with the intervention was the most commonly assessed outcome, but this concept lacks theoretical underpinning and may not be a good indicator of intervention quality (54). The use of global satisfaction ratings is particularly susceptible to the “halo effect” whereby raters overestimate performance with global impressions influencing responses to specific items. In interventions involving health professionals, patients may also report on the clinicians’ interpersonal skills rather than the clinicians’ technical competence or the intervention’s usefulness (55, 56). It is hoped that the emergence of standardized tools such as the EORTC QLQ-INFO25 will encourage the assessment of information provision and related constructs, thus providing greater understanding of whether interventions achieve real change and allowing comparison across studies of intervention effects. If satisfaction ratings are to be used, it is recommended that surveys emphasize that the ratings will be used to improve the intervention (rather than merely to evaluate it) and include more items assessing specific aspects of the intervention, rather than using a global rating. Both of these suggestions have been shown to reduce the impact of the halo effect (56).

The number of studies conducted with this population seemed to increase over time, with several conducted during the most recent decade. This is promising and may reflect a renewed interest in improving the treatment outcomes for patients with brain tumors, and also the encouragement provided through successfully conducted previous studies. Most studies, however, employed research designs that resulted in either high risk of bias or inability to assess risk of bias, lacking a published study protocol and a control group in most instances. Although the nature of the interventions mean that it would not be possible to blind participants to study outcomes, blinding of assessors would be feasible. Greater specification of analysis methods (for example, if intention-to-treat analysis was carried out) is also needed. Finally, none of the included studies investigated whether intervention efficacy was affected by patients’ cognitive status, despite cognitive impairment being a common issue in this population (29, 31, 32). This should be considered in future studies.

### Strengths and limitations of this review

To the best of our knowledge, this is the first systematic review of interventions to improve information provision for adult primary brain tumor patients and their caregivers. Strengths of this systematic review include the extensive search of the literature in multiple databases, the inclusion of publications written in languages other than English, and the assessment of risk of bias of included studies. However, due to the limited number of studies, heterogeneity in interventions and methods, and inadequate reporting of data for some studies, we were unable to statistically pool the study results to determine the relative benefit of different interventions. Further work is necessary to determine the most effective intervention components and most appropriate timing for intervention delivery, as well as the effect of interventions on more distal outcomes such as quality of life, treatment adherence, or survival.

## Conclusion

This systematic review showed that interventions with an educational component improve information provision and knowledge for adults with brain tumors, their families, and caregivers. Furthermore, satisfaction with these interventions was high. Although these results are promising, future efficacy and effectiveness trials with rigorous study designs are needed, particularly to determine the most useful intervention components and to understand if certain subgroups of the population are differentially affected.

## Conflict of Interest Statement

The authors declare that the research was conducted in the absence of any commercial or financial relationships that could be construed as a potential conflict of interest.
